# Supplementary material for: Population-based cohort across stroke life course in India-The NIMHANS-NH-SKAN stroke project: A study protocol
Source: PLoS One. 2024 Oct 2;19(10):e0310309. doi: 10.1371/journal.pone.0310309 (PMC11446417; doi:10.1371/journal.pone.0310309)
Supplement: S1 Table — (DOCX) [file pone.0310309.s001.docx]

**Supporting information:**

**S1 Table: Definitions proposed to ascertain exposures & outcomes for stroke-free period, stroke and post-stroke period**

| **S. no** | **Risk factor groups** | **Tools or measurements** | **Definitions** |
| --- | --- | --- | --- |
| 1 | Obesity/Overweight | Anthropometric measures- Height and weight measurement using a stadiometer and a weighing scale.  Waist and hip circumference – using a measuring tape | **Body Mass Index (BMI)**: using Quetelet’s Index. (BMI=Weight in Kg/Height in m²)-according to the revised guidelines for India(1).  Categorized as:   - Underweight (BMI<18.5 kg/m^2^), - Normal or lean (BMI 18.5–22.9 kg/m^2^), - Overweight (BMI 23.0 –24.9 kg/m^2^) and - Obese (BMI≥25 kg/m^2^)   **Waist – Hip circumference** (2) categorized:   - Waist-Hip Ratio (WHR)- For male >0.9 and for female >0.85 |
| 2 | Mental health/ Stress | Those who are clinically diagnosed as having depression/ anxiety/stress by a psychiatrist (based on medical records) and/or by administering an appropriate diagnostic or screening scale/instrument.  Screening for Generalised Anxiety Disorder using Generalized anxiety disorder-7 (GAD-7) (3) .  Screening for depression using Patient Health Questionnaire (PHQ-9) (4).  Perceived Stress Scale (PSS-10) (5) for measuring perception of stress.  Workplace stress – Using tool to assess and classify work–related stress (TAWS-16) and symptoms suggestive of work stress (6). | **Generalized Anxiety Disorder (GAD-7)**: a seven-question tool that measures the severity of generalized anxiety disorder (GAD) and categorized through scores:   - Minimal anxiety (0–4) - Mild anxiety (5–9) - Moderate anxiety (10–14) - Severe anxiety (15–21)   **Patient Health Questionnaire-9 (PHQ-9):** 9 item screening tool for assessment of severity of depressive symptoms categorized through scores:   - Minimal depression (0–4) - Mild depression (5–9) - Moderate depression (10–14) - Moderately severe depression (15–19) - Severe depression (20-27)   **Perceived Stress Scale (PSS-10):** 10- item questionnaire categorized as:   - Low stress (0-13). - Moderate stress (14-26). - High perceived stress (27-40).   **TAWS-16:** 16 item questionnaire Tool to assess and classify work–related stress (TAWS-16). Administered only for those respondents who are working. It is categorized as:   - Work stress present (Score >48) - Mild stress (cut of 60%) of maximum possible score (Score between 48-59) - Moderate work stress (61-75% of maximum) (Score between 60-73) - Severe work stress (>75% of maximum) (Score >73)   **Symptoms suggestive of work stress** is a 16-item questionnaire categorized as:   - symptoms suggestive of work stress (score >16) - Mild experience of symptoms of work stress (score between 16-29) - Moderate experience of symptoms of work stress (score between 30-36) - Severe symptoms of work stress (score >36) |
| 3 | Non-Communicable disease | Those who are diagnosed with anyone or more of Diabetes Mellitus, Hypertension, Dyslipidemia and Coronary artery disease | **Diabetes:** 8-hr fasting capillary/venous blood glucose ≥126 mg/dl or 2h venous post-glucose value ≥220 mg/dl (assessed using hexokinase method) and/ taking medications for diabetes (7,8)  **Impaired fasting glucose:** Fasting capillary/venous blood glucose ≥110 and <126 mg/dl (assessed using hexokinase method) (9,10) |
|  |  |  | **Hypertension**(11)   \|  \| Systolic \| Diastolic \| \| --- \| --- \| --- \| \| Normal blood pressure \| <120 mm Hg \| < 80 mm Hg \| \| Elevated blood pressure \| 120-129 mm Hg \| < 80 mm Hg \| \| Stage 1 Hypertension \| 130-139 mm Hg \| 80-89 mm Hg \| \| Stage 2 Hypertension \| >140 mm Hg \| > 90 mm Hg or taking treatment \|   **Hypercholesterolemia:** Serum cholesterol levels ≥200 mg/dl (≥5.2 mmol/liter) (using enzymatic, Colorimetric method) or on documented treatment for hypercholesterolemia.  **Hypertriglyceridemia:** Serum triglyceride levels ≥150 mg/dl (≥1.7 mmol/liter) (using enzymatic, Colorimetric method) or on documented treatment for hypertriglyceridemia.  **Low High-Density Lipoprotein (HDL) Cholesterol:** Serum HDL cholesterol levels <40 mg/dl (<1.04 mmol/liter) for men and <50 mg/dl (<1.3 mmol/liter) (using homogenous enzymatic, Colorimetric method) for women.  **High Low-Density Lipoprotein (LDL) Cholesterol:** Serum LDL cholesterol levels ≥130 mg/dl(using homogenous enzymatic, Colorimetric method). (12) |
|  |  |  | **Coronary Artery Disease (CAD):** History of documented myocardial infarction and/or medical therapy or revascularization for coronary artery disease (CAD) and/or Electro Cardiogram (ECG) changes such as Q-wave changes (Minnesota codes 1-1-1 to 1-1-7) and/ or ST segment depression (Minnesota codes 4-1 to 4-2) diagnosed and documented by a trained physician/cardiologist (13). |
| 4 | Substance use | Study subjects who use any one or more of tobacco (smoke and smokeless forms), alcohol and other substances.  Smoking Dependence-Using Fagerström Nicotine Dependence scale (FTND) (15)  Smokeless tobacco Dependence: Fagerström Nicotine Dependence scale– Smokeless tobacco (FTND-ST)  Alcohol use: AUDIT (alcohol use disorder identification test).  Drug use – Based on CAGE AID questionnaire- a 4-item scale | **Current smoker**: who at the time of survey, either smokes any tobacco product daily or occasionall*y* (14).  **Smoking Dependence** - FTND (15)scores categorized as-   - Low dependence:1-2 - Low to moderate dependence:3-4 - Moderate dependence:5-7 - High dependence:8+   **Current smokeless tobacco user**: who at the time of the survey uses any form of smokeless tobacco either daily or occasionally within the past 30 days. (16)  **Smokeless tobacco Dependence** : FTND-ST (17) - scores ranging from-:   - Low to moderate dependence: ≤ 4 - Significant dependence: ≥5   ***Current Alcohol*** user means those who have consumed a drink containing alcohol in the last 12 months (18).  **Alcohol use:** AUDIT (alcohol use disorder identification test). The range of possible scores is from 0 to 40 (19)   \| Range \| Audit score \| \| --- \| --- \| \| Low-risk \| 0-7 \| \| Hazardous or Harmful \| 8-14 \| \| Alcohol dependent \| 15+ \|   **Drug use** – Based on CAGE AID questionnaire which is a 4-item scale. A total score of 2 or greater is considered clinically significant. (20) |
| 5 | Nutrition | The questions consist of quantity of all items consumed by the participant’s family based on monthly utilization of cereals including millets, pulses, vegetables, roots and tubers, oils, sugars, salt and fruits. | Average consumption of each of cereals, pulses, oils, sugars and salt.  Unhealthy diet: Those consuming less than five standards serving of fruits or vegetables in a typical day (21). |
| 6 | Physical inactivity | Estimated using Global Physical Activity Questionnaire (GPAQ): GPAQ consists of 16 questions designed to estimate an individual’s level of physical activity in 3 domains (work, transport and leisure time) and time spent in sedentary behavior. | Physical inactivity: Individuals achieving less than 600 MET-min/week (based on any combination of walking/cycling, moderate-intensity or vigorous intensity activities) (22) |
| 7. | Covid-19 infection | Questions related to their | Those tested positive by Polymerase Chain Reaction or Antigen tests. |
| 8. | Covid-19 vaccination | Questions related to Covid-19 vaccination and number of doses. | Those received covid-19 vaccination with proof/documentary evidence |
| 9. | Insomnia | Screened using Global Sleep Assessment Questionnaire (GSAQ): GSAQ consists of 11 questions designed to assess sleep quality. | Participants answering “USUALLY” or “ALWAYS” to question “Did you have difficulty falling asleep, staying asleep, or feeling poorly rested in the morning?” in Global Sleep Assessment Questionnaire. |
| 10. | Obstructive sleep apnea | Screened using Global Sleep Assessment Questionnaire (GSAQ): GSAQ consists of 11 questions designed to assess sleep quality. | Participants answering “usually” or “always” to question “Did you snore loudly?” or “Did you hold your breath, have breathing pauses, or stop breathing in your sleep?” in Global Sleep Assessment Questionnaire. |
| 11. | Risk of cell phone addiction | Screened using Global Sleep Assessment Questionnaire (GSAQ): GSAQ consists of 11 questions designed to assess sleep quality. | Participants answering “YES” to more than three questions of the standard to assess risk of cell phone addiction. |
| 12. | Restless leg syndrome | Screened using Global Sleep Assessment Questionnaire (GSAQ): GSAQ consists of 11 questions designed to assess sleep quality. | Participants answering “usually” or “always” to question “Did you have repeated rhythmic leg jerks or leg twitches during your sleep?” |
| **Outcome** | | | |
|  | Stroke | - Ischemic Stroke - Hemorrhagic Stroke - Subarachnoid hemorrhage - Cerebral venous thrombosis | As per specialist diagnosis or medical record |
|  | Survival | Status of the respondent | Whether the individual is living or deceased |
|  | Disability | Disability is measured using Modified Rankin Scale (mRS).(23) It is a 7-point disability scale with possible scores ranging from 0 to 7. mRS measures the degree of disability in patients who have had a stroke. | mRS is categorized as:   - - - - No symptoms at all (score “0”)       - No significant disability: despite symptoms, able to carry out all usual duties and activities (score “1”)       - Slight disability: unable to perform all previous activities but able to look after own affairs without assistance (score “2”)       - Moderate disability: requiring some help but able to walk without assistance (score “3”)       - Moderately severe disability: unable to walk without assistance and unable to attend to own bodily needs without assistance (score “4”)       - Severe disability: bedridden, incontinent and requiring constant nursing care and attention (score “5”)       - Death (score “6”) |
|  | Quality of Life | SSQOL (Stroke-specific quality of life) (24) measures patient-centered outcomes such as functional status and health-related quality of life. Items are rated on a 5-point [Likert scale](https://strokengine.ca/en/glossary/likert-scale/). | SSQOL- The total score ranges from 49 to 245, with higher scores indicating a better quality of life. |

References:

1. Misra A, Chowbey P, Makkar BM, Vikram NK, Wasir JS, Chadha D, et al. Consensus statement for diagnosis of obesity, abdominal obesity and the metabolic syndrome for Asian Indians and recommendations for physical activity, medical and surgical management. J Assoc Physicians India. 2009 Feb;57:163–70.
2. World Health Organization. Waist Circumference and Waist–Hip Ratio: Report of a WHO Expert Consultation.: Geneva: World Health Organization; 2008.
3. Spitzer RL, Kroenke K, Williams JBW, Löwe B. A Brief Measure for Assessing Generalized Anxiety Disorder: The GAD-7. Arch Intern Med. 2006 May 22;166(10):1092–7.
4. Patient Health Questionnaire (PHQ-9 & PHQ-2) [Internet]. [cited 2024 Jan 29]. Available from: <https://www.apa.org/pi/about/publications/caregivers/practice-settings/assessment/tools/patient-health>
5. The PHQ-9 - PMC [Internet]. [cited 2024 Jan 30]. Available from: <https://www.ncbi.nlm.nih.gov/pmc/articles/PMC1495268/>
6. Roy R, Sukumar GM, Philip M, Gopalakrishna G. Face, content, criterion and construct validity assessment of a newly developed tool to assess and classify work–related stress (TAWS– 16). PLOS ONE. 2023 Jan 6;18(1):e0280189.
7. WHO technical specifications for blood glucose meter [Internet]. [cited 2024 Jan 29]. Available from: https://www.who.int/publications/m/item/who-technical-specifications-for-blood-glucose-meter
8. Alberti KG, Zimmet PZ. Definition, diagnosis and classification of diabetes mellitus and its complications. Part 1: diagnosis and classification of diabetes mellitus provisional report of a WHO consultation. Diabet Med J Br Diabet Assoc. 1998 Jul;15(7):539–53.
9. Mohan V, Kaur T, Anjana RM, Pradeepa RG. ICMR-India DIABetes [INDIAB] Study Phase 1Final Report (2008-2011). Indian Council of Medical Research;2018.
10. National Programme for Prevention and Control of Cancer, Diabetes, Cardiovascular Diseases & Stroke (NPCDCS) Operational Guidelines Revised (2013-2017). Directorate General of Health Services, Ministry of Health & Family Welfare, Government of India; 2013.
11. Performance Measures Hypertension | ACP Online [Internet]. [cited 2024 Jan 29]. Available from: <https://www.acponline.org/clinical-information/performance-measures/clinical-topic/Hypertension>
12. Cleeman J. ATP III Guidelines At-A-Glance Quick Desk Reference
13. Clinical values of resting electrocardiography in patients with known or suspected chronic coronary artery disease: a stress perfusion cardiac MRI study - PMC [Internet]. [cited 2024 Jan 29]. Available from: https://www.ncbi.nlm.nih.gov/pmc/articles/PMC8714441/
14. Tata Institute of Social Sciences (TISS), Mumbai and Ministry of Health and Family Welfare, Government of India. Global Adult Tobacco Survey GATS 2 India 2016-17. New Delhi: Ministry of Health and Family Welfare;2018.
15. Heatherton TF, Kozlowski LT, Frecker RC, Fagerström KO. The Fagerström Test for Nicotine Dependence: a revision of the Fagerström Tolerance Questionnaire. Br J Addict. 1991 Sep;86(9):1119-27.
16. Smokeless Tobacco Use in the United States | CDC [Internet]. [cited 2024 Jan 29]. Available from: https://www.cdc.gov/tobacco/data_statistics/fact_sheets/smokeless/use_us/index.ht
17. Ebbert JO, Patten CA, Schroeder DR. The Fagerström Test for Nicotine Dependence-Smokeless Tobacco (FTND-ST). Addict Behav. 2006 Sep;31(9):1716-21.
18. Current drinkers [Internet]. [cited 2024 Jan 29]. Available from: https://www.who.int/data/gho/indicator-metadata-registry/imr-details/3680.
19. Scoring the AUDIT [Internet]. [cited 2024 Jan 29]. Available from: https://auditscreen.org/about/scoring-audit
20. CAGE Questionnaire: Purpose, Questions, After Results [Internet]. [cited 2024 Jan 29]. Available from: <https://www.verywellhealth.com/cage-questionnaire-5216479>
21. Healthy diet [Internet]. [cited 2024 Jan 29]. Available from: <https://www.who.int/news-room/fact-sheets/detail/healthy-diet>
22. Global physical activity questionnaire (GPAQ) [Internet]. [cited 2024 Jul 27]. Available from: <https://www.who.int/publications/m/item/global-physical-activity-questionnaire>
23. Outcomes Validity and Reliability of the Modified Rankin Scale: Implications for Stroke Clinical Trials | Stroke [Internet]. [cited 2024 Jan 29]. Available from: <https://www.ahajournals.org/doi/10.1161/01.str.0000258355.23810.c6>
24. Development of a Stroke-Specific Quality of Life Scale | Stroke [Internet]. [cited 2024 Jan 29]. Available from: https://www.ahajournals.org/doi/full/10.1161/01.STR.30.7.1362
